# Maximising follow-up participation rates in a large scale 45 and Up Study in Australia

**DOI:** 10.1186/s12982-016-0046-y

**Published:** 2016-04-14

**Authors:** Adrian Bauman, Philayrath Phongsavan, Alison Cowle, Emily Banks, Louisa Jorm, Kris Rogers, Bin Jalaludin, Anne Grunseit

**Affiliations:** Prevention Research Collaboration, Level 6 Charles Perkins Centre D17, Sydney School of Public Health, University of Sydney, Sydney, NSW 2006 Australia; Sax Institute, University of Technology, PO Box K617, Haymarket, NSW 1240 Australia; National Centre for Epidemiology and Population Health, Australian National University, Building 62, Acton, ACT 0200 Australia; Centre for Big Data Research in Health, Faculty of Medicine, University of New South Wales, Sydney, NSW 2052 Australia; The George Institute for Global Health, PO Box M201, Missenden Road, Camperdown, NSW 2050 Australia; School of Public Health and Community Medicine, University of New South Wales, Sydney, Australia; Centre for Research, Evidence Management and Surveillance, Locked Bag 7279, Liverpool, NSW 1871 Australia

**Keywords:** Epidemiological studies, Follow-up, Response rates, Recruitment strategies

## Abstract

**Background:**

The issue of poor response rates to population surveys has existed for some decades, but few studies have explored methods to improve the response rate in follow-up population cohort studies.

**Methods:**

A sample of 100,000 adults from the 45 and Up Study, a large population cohort in Australia, were followed up 3.5 years after the baseline cohort was assembled. A pilot mail-out of 5000 surveys produced a response rate of only 41.7 %. This study tested methods of enhancing response rate, with three groups of 1000 each allocated to (1) receiving an advance notice postcard followed by a questionnaire, (2) receiving a questionnaire and then follow-up reminder letter, and (3) both these strategies.

**Results:**

The enhanced strategies all produced an improved response rate compared to the pilot, with a resulting mean response rate of 53.7 %. Highest response was found when both the postcard and questionnaire reminder were used (56.4 %) but this was only significantly higher when compared to postcard alone (50.5 %) but not reminder alone (54.1 %). The combined approach was used for recruitment among the remaining 92,000 participants, with a resultant further increased response rate of 61.6 %.

**Conclusions:**

Survey prompting with a postcard and a reminder follow-up questionnaire, applied separately or combined can enhance follow-up rates in large scale survey-based epidemiological studies.

## Background

The majority of population-based cohort studies rely on individuals’ willingness to give their time and effort to participate. In recent decades, many epidemiological cohort studies have experienced declining participation or response rates, raising concerns about the usefulness of cohort findings for research and practice [1, 2] and posing practical and methodological challenges. Chief among these is identifying strategies to maximising response rates for assuring external validity of the findings. Maximising participation rates at recruitment and during the follow-up period is crucial, given that the characteristics of non-responders may show a socio-economic gradient [3] or higher mortality than survey responders [4].

For many decades, researchers have attempted to increase response rates to postal questionnaires. An early systematic review [5] and a meta-analysis [6] identified factors such as increased contacts and reminders, pre-notification by mail, credible sources of the study and perceived relevance of the topic can influence study participation rates. A Cochrane review summarised strategies to increase postal questionnaire responses, and identified incentives, personalising follow up, and follow-up reminder questionnaires as among strategies that effectively increased response rates [7]. Despite the plethora of research, most studies were small-to-mid sized (less than 1000 people approached) [e.g. 9], and most assessed recruitment to enrol in a study or trial [8, 9]. A more recent report described recruitment to an epidemiological cohort [10] and another identified that different population subgroups responded differently to multiple mail-out reminders [11].

Retention is a further concern within cohort studies, with loss to follow-up as a recognised problem that may cause greater bias than initial recruitment. In recent years, researchers have focused effort on baseline recruitment, but few studies have tested different recruitment strategies to increase subsequent retention for follow-up in large population-based cohorts. Strategies to increase retention have been trialled in HIV prevention studies in younger adults [12] and in one cardiac trial, home visiting increased study retention [13]. Reminder letters, together with the provision of stamped or business-reply envelopes and delivery of a newsletter have also influenced follow-up response rates [14]. An older adult trial for neck injuries trialled a £5 incentive to increase four- and eight-month questionnaire returns, and achieved higher responses, but at a cost of £67 per additional questionnaire received [15]. A recent systematic review found incentives increased retention rates (increasing with higher values) in population-based cohort studies [16]. Overall, the research to date has used similar strategies for increasing recruitment and achieving continuing participation during the follow-up period. Existing evidence withstanding, little research has addressed retention in epidemiological cohort studies, using different postal pre-notification (advance notice) and reminder approaches to maximise follow-up questionnaire responses sent several years after the baseline. Additionally, it is important to consider that the factors influencing participation and retention may be different; that is, it is possible that different mechanisms may operate once a participant has consented and expect to be in a cohort study over an extended period.

This study focuses on a 3.5-year follow up of a large sub-sample of the 45 and Up Study Cohort [17]. The follow up sub-study assesses social, environmental and economic factors (SEEF) and subsequent health outcomes, as well as changes in several baseline attributes from the 45 and Up Study. The recruitment goal of the SEEF Study was to re-contact the first 100,000 participants from the baseline 45 and Up Study and invite them to take part in the follow-up sub study (i.e. the SEEF Study). Recruitment for the SEEF Study was challenging, as it involved a large sample, and therefore low-cost methods for optimal follow-up strategies were required. This paper evaluates the strategies used to maximise the response rate to the SEEF Study and presents cost analysis of the strategies, to inform subsequent 45 and Up Study follow-up studies and large cohort studies elsewhere.

## Methods

### Study population

The 45 and Up Study assembled a large (n = 267,153) sample of adults aged 45 up to 110 years living in the state of New South Wales, Australia, with the objective of following their health status. Baseline data were collected between 2006 and 2009, and covered previous and current health status, behavioural risk factors and health service utilisation [18]. Information on study design, sampling method and baseline cohort profile is reported elsewhere [17]. Although the 45 and Up Study baseline response rate was modest (18%), representativeness is not essential in cohort studies and observed cross sectional exposure-outcome relationships were similar to state-based surveillance systems that reported much higher response rates [19–21].

### Study design and data collection

**Fig. 1:**
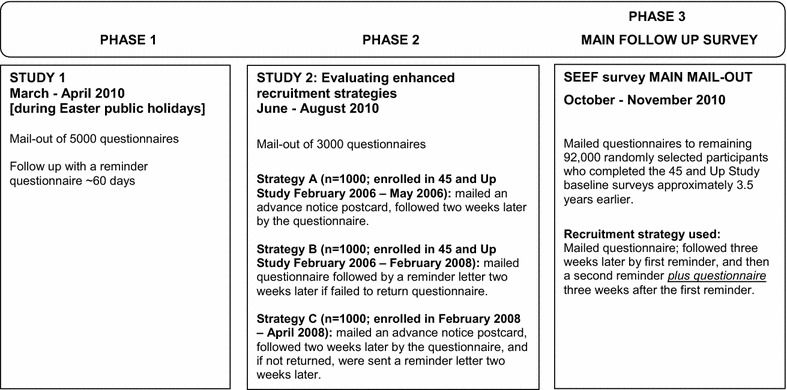
Phases of testing different recruitment strategies

The first 100,000 people to join the 45 and Up Study, excluding those deceased by 2010 or those already recruited to participate in other follow-up sub studies (3.6 % of baseline) were eligible to join the SEEF Study. Figure 1 shows the key phases of the SEEF Study, specifically Phase 1 pilot recruitment; Phase 2 evaluated three different recruitment strategies and; Phase 3 the main follow-up data collection using the recruitment strategies identified to be most effective in yielding the higher response rate based on Phase 2 results.

**Fig. 2:**
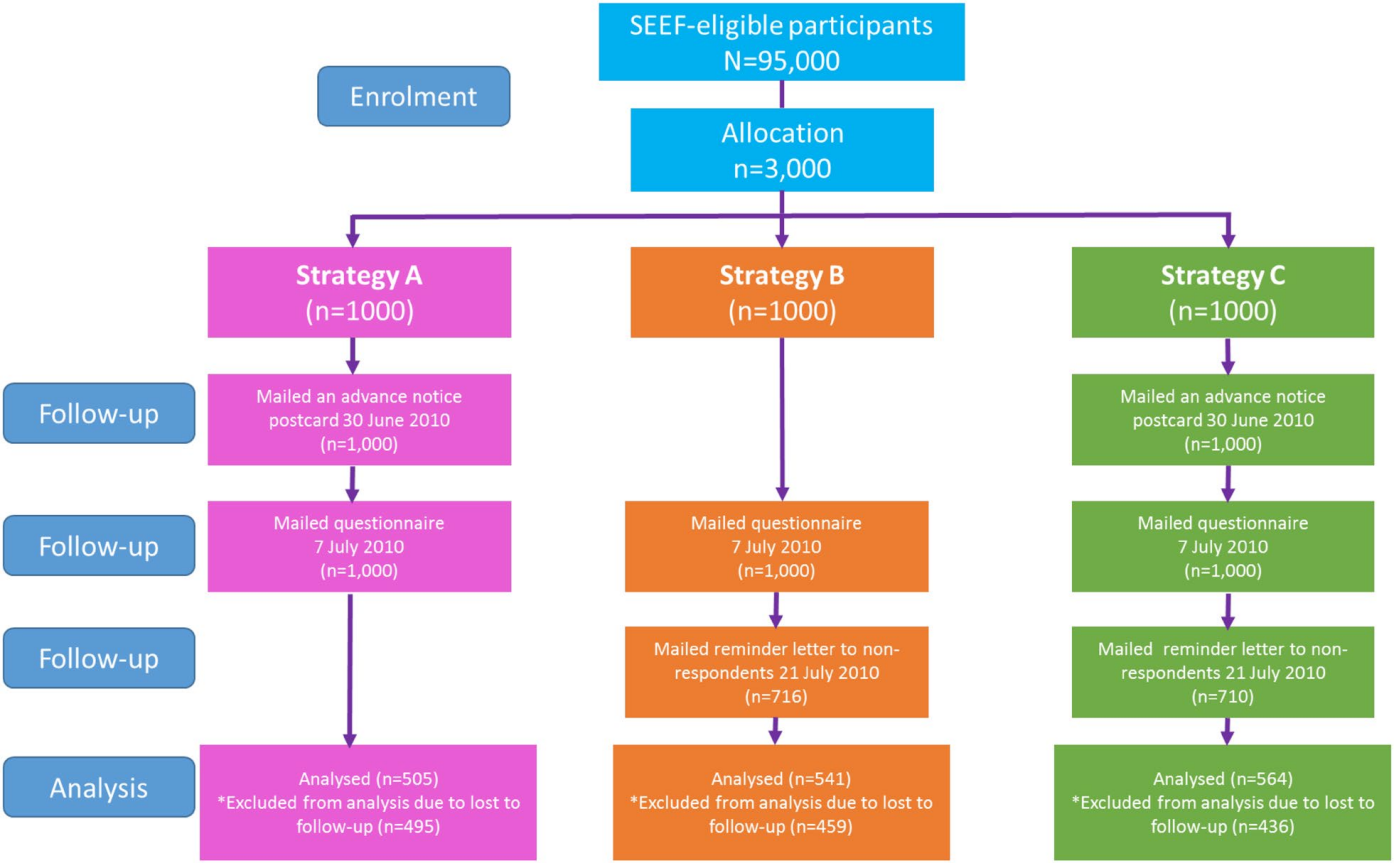
Flow diagram of three mail-out enhanced recruitment strategies [Study 2]

In Phase 1, a sample of 5000 participants from the 45 and Up Study were randomly selected from the 100,000 participants identified as eligible to take part in the SEEF Study, and mailed a questionnaire in March 2010 (Study 1), with a 60-day reminder. This phase occurred during the Easter holiday period. Due to the poor response rate, a decision was made to develop three different strategies to improve recruitment and their effectiveness assessed (Study 2) in Phase 2. Using the SAS statistical program [22], three thousand participants were randomly sampled from the remaining 95,000 SEEF-eligible participants and allocated to three groups of 1000 participants each according to date of joining the 45 and Up Study (Fig. 1), to receive between June and August 2010, either (Fig. 2):

1. an advance notice postcard followed by the SEEF questionnaire 2 weeks later (Strategy A);
2. a SEEF questionnaire followed by a reminder letter 2 weeks later (Strategy B), or;
3. an advance notice postcard, followed 2 weeks later by the SEEF questionnaire, followed another 2 weeks later by a reminder letter (Strategy C).

A statistician blinded to participant identity performed the randomisation sequence and was not involved in preparing the SEEF Study questionnaire packages for outward mailing; a logistics agency was contracted to administer the mail-put of questionnaire packages and staff were not informed of the allocation.

The 8-page questionnaire included a subset of repeated questions from the baseline 45 and Up survey (http://www.45andup.org.au) and additional questions on a range of social, psychological, economic and environmental factors associated with health and wellbeing. Questionnaire completion time varied from 30 to 45 min. Both the one-page reminder letter and the postcard provided brief information about the SEEF Study, an invitation to participate and were signed by the 45 and Up Study Scientific Director. Participants were provided with reply-paid envelopes to return their completed questionnaires.

In the final Phase 3, a combined strategy was used to recruit among the remaining 92,000 between late 2010 and early 2011; a questionnaire was mailed out, followed at 3 weeks by a reminder letter to non-responders. After a further 3 weeks, a second reminder with a questionnaire was mailed out to non-responders; this latter component was an adaptation of the Phase 2 study.

The study received ethical approval from the University of Sydney Human Research Ethics Committee (Ref No. 10-2009/12187); and the University of New South Wales Human Research Ethics Committee granted ethics approval for the 45 and Up Study (Ref No. HREC 05035).

### Statistical analyses

The main outcome was participation in the SEEF Study, consisting of receipt of a completed SEEF questionnaire and signed consent form. Response rates were calculated as: those participating in SEEF divided by (those eligible for SEEF, minus participants whose letters were returned to sender, and who were not further followed). Response rates were examined overall, and by sex and age group, with age categories as 45–64, and 65 years and older.

In order to test the impact of each method of enhancement, we used generalised linear models with a log link and binomial distribution [23] with survey participation as the outcome, and condition (postcard (Strategy A), reminder (Strategy B), and reminder plus postcard (Strategy C) as the independent variable with no enhancement (Study 1) as the reference category. Planned contrasts between the three enhancement conditions were performed. Results are reported as prevalence ratios (PR). All analyses were conducted using Stata 13.0 [24] and 0.05 the threshold for statistical significance.

Completed SEEF hard-copy questionnaire forms were scanned with optical character recognition software and compiled into an indexed SAS dataset [22]. Costs of each recruitment strategy were computed, by summing printing and postage costs for each strategy, and assessing the cost per questionnaire received.

## Results

The Study 1 pilot of 5000 mailed questionnaires had a 30-day response rate of 41.7 %. This response rate was similar by gender, and by age group (Table 1). In the three subsequent intensive mail-out conditions in Study 2, the 14-day response rates ranged from 28 to 29 %; Strategy A returned 282 completed questionnaires, Strategy B returned 284 questionnaires and Strategy C returned 290 questionnaires (Fig. 2). Overall response rates were incrementally greater with increasing intensity of follow-up procedures. A response of 50.5 % was achieved with the pre-questionnaire postcard only (Strategy A), 54.1 % responding to only the post questionnaire reminder (Strategy B), and 56.4 % responding to both the pre-postcard and post questionnaire reminder (Strategy C). Overall, the intensive conditions of Study 2 achieved a 53.7 % combined response rate (95 % CI 51.9–55.5), which was significantly higher than the 41.7 % (40.3–43.5 %) response rate in Study 1.

**Table 1:** Recruitment strategies for Study 1 mail-out pilot and Study 2 of three enhanced mail-out strategies

Study and intervention arm of study	Date mailed out	Cost per unit received ($AU)	Responses 30 days after questionnaire mail-out	Overall RR (%) and 95 % CI	Gender				AGE			
					Males		Females		Aged 45–64 years		Aged 65+ years	
					Responded	RR	Responded	RR	Responded	RR	Responded	RR
*Study 1 pilot*												
5000 Questionnaire	Questionnaire (Q) sent 24 March 2010	$4.19	2084	41.7 %* [40.3–43.5]	961	41.2 % [39.2–43.2]	1123	42.1 % [40.2–43.9]	1233	42.6 % [40.8–44.4]	851	40.4 % [38.3–42.5]
*Study 2 enhanced mail-out*												
*Strategy A* 1000 Postcard + Questionnaire	Postcard 30 June 2010 + Q 7 July 2010	$3.13	505	50.5 %** [47.4–53.5]	229	48.8 % [44.3–53.3]	276	52.0 % [47.7–56.2]	315	52.2 [48.2–56.2]	190	48.0 % [43.1–52.9]
*Strategy B* 1000 Questionnaire + Reminder	Q 7 July 2010 + Reminder 21 July 2010	$3.20	541	54.1 % [51.0–57.2]	278	52.4 % [48.1–56.6]	263	56.1 % [51.6–60.5]	330	55.6 % [51.6–59.6]	211	51.8 % [47.0–56.7]
*Strategy C* 1000 Postcard + Questionnaire + Reminder	Postcard 30 June 2010 + Q 7 July 2010 + Reminder 21 July 2010	$3.78	564	56.4 % [53.3–59.4]	242	60.0 % [55.2–64.7]	322	53.9 % [49.9–57.9]	350	57.7 % [53.7–61.5]	214	54.5 % [49.5–59.3]

Q questionnaire mailed out; RR response rate (computed as at 30 days from sending the invitation/questionnaire pack)

* Study 1 different to all arms of Study 2, p < 0.05; ** Unadjusted (Strategy A) versus (Strategy C) p = 0.004; (Strategy A) versus (Strategy B) p=0.053

Compared to the pilot mail-out to 5000 participants in Study 1, the effect of sending a postcard when there is no reminder was 1.21 times as effective (95 % CI 1.13–1.30). Using a postcard prompt also resulted in increased likelihood of survey response (PR = 1.30, 95 % CI 1.22–1.39), as did if both a postcard and a reminder were sent out (PR 1.35, 95 % CI 1.27–1.44) (data not shown). The contrast analyses showed that using a reminder or a postcard were comparable, and adding a postcard to a reminder did not significantly improve the response rate compared to only sending a reminder. However, the combination of postcard and reminder was significantly better than postcard alone (PR 1.12, 95 % CI 1.03–1.21).

The costs of the recruitment per person were highest for Study 1, and similar for each strategy in Study 2. The most expensive enhanced recruitment approach was both reminder and postcard, which cost around $0.65 more than a postcard alone, per person recruited.

For the main SEEF Study mail-out (Phase 3 of this study), of the remaining 92,000 SEEF-eligible participants, a response rate of 61.6 % (95 % CI 61.3–61.9 %) was obtained, significantly higher than either Study 1 or Study 2. The recruitment approach for the main survey was an adaption of the Study 2 strategies and used a combination of mail-out, followed at 3 weeks by a reminder letter to non-responders, followed by a second reminder plus a questionnaire to the remaining non-responders.

## Discussion

Efficient methods for recruiting and following up existing participants are essential in large-scale prospective studies if they are to remain invaluable in epidemiological research. In this paper, we report the effectiveness of three enhanced recruitment strategies to improve follow up response rates in a social epidemiological study. The results of this study show that more intensive strategies can improve follow-up response rate. Among the three strategies trialled, the pre-postcard, mailed questionnaire and reminder mailed letter at 2 week intervals had the highest response rate. However, having any kind of prompt alone or in combination appears to be better than having no prompt, consistent with other studies using a pre-notification (advance notice) or reminders [3]. Another epidemiological study also found similar follow-up response rates using reminder strategies based on style of return envelope and delivery of a newsletter [14]. The cost analysis did not define a clear benefit, but all additional costs were cheaper per unit survey than in other research [15].

Overall, the SEEF study across Study 1, Study 2, and the subsequent adaptation that involved also sending a reminder questionnaire at follow-up resulted in 60,404 in the complete data set, indicating just over a 60 % response rate. This response rate represents a reasonable return rate at an acceptable cost, without compromising the scientific integrity of the study. Further, these results are within the ranges obtained by large epidemiological cohort studies [25]. However, age differentials were observed in this study, with lower response among those aged over 65 years. Such age-related declines in response rates during follow-up period are expected in older adult cohort studies as a result of attrition due to deaths and disability [2, 26].

Our study found some gender differences to response based on the strategies used. Women receiving a questionnaire plus a reminder were more likely than their counterparts receiving a postcard and a reminder, separately or in combination, to respond to the survey. For men, a combination of pre-notification and reminders generated a substantially higher response rate in this group compared to women, suggesting that male participants may respond better to frequent prompts and reminders. In other research, frequent reminder prompts to male participants also indicated a positive effect on retention rates [16]; although to our knowledge no study had specifically looked at gender differentials to repeat contacts. Further research will need to investigate the impact of frequency and recruitment methods to boost follow-up response rates among men, a group widely known to be less likely to take part in research studies.

A major strength of this study is its sample size, with 8000 population-based individuals approached in the two studies. The literature to date shows that studies of similar scope and size are rarely conducted (typically less than a total of 1000). The cost analysis is another strength, indicating that the additional costs associated with the most intensive strategy (Strategy C) is marginal when considering the higher response rate generated by the strategy. Our study focused on postal questionnaires and did not examine ethnic differential effects which may limit its generalisability to studies involving large non-English speaking participants. This study did not model other factors that could predict participation (e.g. age, gender, health profile, timing of joining the baseline 45 and Up Study). We did stratify the analysis by gender and age, and found response rate differentials across these factors. It is possible that the lower response among the oldest age group (65+ years) may have been the result of natural attrition due to the higher risk of morbidity or mortality among this older age group. Due to the sequential nature of the group allocation to the three recruitment strategies, it is also plausible that the time since last contact may affect the response rates. We acknowledge that our study is further limited by the lack of description of the sub-samples which were targeted by the different strategies, as the varying response rates may be related to the socio-demographic and health-related characteristics.

While this study shows that any kind of reminder alone or in combination is better than having no reminder (Study 1), we cannot state in absolute term which of the three enhanced recruitment strategies (Study 2) is better than another. Further, the pilot recruitment phase occurred during Easter and therefore some of the improvements noted in the following two phases may be attributed to recruiting outside a major public holiday period.

## Conclusions

This study explored alternate methods for increasing follow-up response rates in a large epidemiological cohort study. The findings indicated that increasing the follow-up intensity resulted in improved response rates. Specifically, we found that the pre-postcard, mailed questionnaire and reminder mailed letter at 2 week intervals alone or in combination were more effective in eliciting a higher response rate than no prompts at all. This study has made an important contribution to maximising the follow-up response rate, and consequently the usefulness of the SEEF Study follow up among older Australian adults. Recruitment and retention, however, will remain a significant challenge for epidemiological cohort studies. Future research in this area needs to consider developing and testing low-cost, innovative recruitment and retention strategies, including investigating potential mechanisms that may influence participation and whether these would vary by gender.

## Abbreviation

SEEF: Social, Environmental and Economic Factors
